# Prognostic Value of Hybrid PET/MR Imaging in Patients with Differentiated Thyroid Cancer

**DOI:** 10.3390/cancers14122958

**Published:** 2022-06-15

**Authors:** Leandra Piscopo, Carmela Nappi, Fabio Volpe, Valeria Romeo, Emanuele Nicolai, Rosj Gallicchio, Alessia Giordano, Giovanni Storto, Leonardo Pace, Carlo Cavaliere, Marco Salvatore, Alberto Cuocolo, Michele Klain

**Affiliations:** 1 Department of Advanced Biomedical Sciences, University Federico II, 80131 Naples, Italy; leandra.piscopo@unina.it (L.P.); c.nappi@unina.it (C.N.); fabio.volpe@unina.it (F.V.); valeria.romeo@unina.it (V.R.); cuocolo@unina.it (A.C.); micheleklain@libero.it (M.K.); 2 IRCCS Synlab SDN, 80143 Naples, Italy; emanuele.nicolai@synlab.it (E.N.); carlo.cavaliere@synlab.it (C.C.); marco.salvatore@synlab.it (M.S.); 3 IRCCS CROB, Referral Cancer Center of Basilicata, 85028 Rionero in Vulture, Italy; rosi.gallicchio@crob.it (R.G.); alessia.giordano@me.com (A.G.); giovanni.storto@crob.it (G.S.); 4 Department of Medicine, Surgery and Dentistry, University of Salerno, 84084 Fisciano, Italy

**Keywords:** differentiated thyroid carcinoma, PET/MR, hybrid imaging, prognosis

## Abstract

**Simple Summary:**

Hybrid positron emission tomography (PET)/magnetic resonance (MR) is an emerging imaging modality with great potential to provide complementary data acquired at the same time, under the same physiological conditions. We evaluated the prognostic value of hybrid ^18^F-fluorodeoxyglucose (FDG) PET/MR in patients with differentiated thyroid cancer (DTC) who underwent total thyroidectomy and radioactive iodine therapy for suspicion of disease relapse. Our findings suggest that hybrid PET/MR imaging may have the potential to improve the information content of one modality with the other and would offer new opportunities in patients with DTC. However, there is need of more clinical studies to understand the additional value of ^18^F-FDG PET/MR in patients with DTC.

**Abstract:**

Background: Hybrid positron emission tomography (PET)/magnetic resonance (MR) is an emerging imaging modality with great potential to provide complementary data acquired at the same time, under the same physiological conditions. The aim of this study was to evaluate the prognostic value of hybrid ^18^F-fluorodeoxyglucose (FDG) PET/MR in patients with differentiated thyroid cancer (DTC) who underwent total thyroidectomy and radioactive iodine therapy for suspicion of disease relapse. Methods: Between November 2015 and February 2017, 55 patients underwent hybrid ^18^F-FDG PET/MR. Assessment of positive MR was made considering all sequences in terms of malignancy based on the morphological T2-weighted features and the presence of restricted diffusivity on diffusion-weighted imaging images and both needed to be positive on the same lesion. Both foci with abnormal ^18^F-FDG uptake, which corresponded to tissue abnormalities on the MR, and tracer accumulation, which did not correspond to normal morphological structures, were considered positive. Results: During follow-up (mean 42 ± 27 months), 29 patients (53%) had disease recurrence. In the Cox univariate regression analysis age, serum Tg level ≥ 2 ng/mL, positive short tau inversion recovery (STIR), and positive PET were significant predictors of DTC recurrence. Kaplan–Meier survival analyses showed that patients with Tg ≥ 2 ng/mL had poorer outcomes compared to those with serum Tg level < 2 ng/mL (*p <* 0.05). Similarly, patients with positive STIR and positive PET had a worst outcome compared to those with negative STIR (*p <* 0.05) and negative PET (*p <* 0.005). Survival analysis performed in the subgroup of 36 subjects with Tg level ≥ 2 ng/mL revealed that patients with positive PET had a worst outcome compared to those with negative PET (*p <* 0.05). Conclusions: Age, serum Tg level ≥ 2 ng/mL, positive STIR, and positive ^18^F-FDG PET were significant predictors of DTC recurrence. However, the serum Tg level was the only independent predictor of DTC. Hybrid PET/MR imaging may have the potential to improve the information content of one modality with the other and would offer new opportunities in patients with DTC. Thus, further studies in a larger patient population are needed to understand the additional value of ^18^F-FDG PET/MR in patients with DTC.

## 1. Introduction

Although differentiated thyroid cancer (DTC) represents the most common endocrine malignancy with an increasing incidence in the last decades, it boasts an excellent prognosis [1,2,3]. On the other hand, there is still a small group of patients who experience a more aggressive form of disease, which is often associated with a poorer outcome. A number of treatment strategies, such as surgical resection of local recurrences, further radioactive iodine (RAI) treatment in select cases, external-beam radiation therapy, and target therapy, may be considered [4,5]. It is conceivable that an optimal diagnostic strategy may be crucial to drive tailored therapeutic approaches. One of the most important diagnostic approaches available in the path of DTC patients is ^18^F-fluorodeoxyglucose (FDG) positron emission tomography (PET)/computed tomography (CT) [6,7,8,9,10]. The American Thyroid Association (ATA) guidelines [4] recommend ^18^F-FDG PET/CT in high-risk patients with measurable thyroglobulin (Tg) and an absence of ^131^I uptake in the whole-body scan (WBS) for both the prognostics and diagnostics of metastatic patients for better management of patients with advanced disease and to evaluate the response to therapy. Based on the greater aggressiveness and dedifferentiation of a positive ^18^F-FDG on PET/CT, it is important to have complementary information from ^131^I uptake at WBS in patients with DTC [11]. Hybrid PET/magnetic resonance (MR) is an emerging imaging modality with great potential to provide important complementary data acquired at the same time, under the same physiological conditions [12,13,14]. Such a tool also has the potential to improve the information content of one modality with the other and offers new opportunities in patients with DTC [15]. Initial attempts to use PET/MR imaging in the evaluation of DTC patients have recently been carried out [16,17,18]. The aim of this study was to retrospectively evaluate the prognostic value of hybrid ^18^F-FDG PET/MR in patients who had already underwent total thyroidectomy and RAI therapy for DTC with suspicion of disease relapse.

## 2. Materials and Methods

### 2.1. Patients

Between November 2015 and February 2017, 55 consecutive patients with DTC previously treated with total or near total thyroidectomy followed by ^131^I ablation were included in the study. In all patients, during routine follow-up, further imaging, such as neck ultrasound, was clinically indicated before empiric ^131^I therapy or surgery, according to the ATA guidelines [4]. All patients underwent hybrid ^18^F-FDG PET/MR. Inclusion criteria were age older than 18 years and the availability of clinical feedback from the patient’s referring physician. Exclusion criteria were pregnancy, blood glucose levels greater than 140 mg/dL (7.77 mmol/L), contraindication to MR imaging, and an inability to tolerate being in the scan due to claustrophobia.

### 2.2. Hybrid PET/MR Imaging

All patients fasted for at least 6 h before imaging. Blood glucose level was assessed with a blood glucose meter (OneTouch Vita; LifeScan, Milpitas, Calif) before imaging to ensure it was less than 140 mg/dL (7.77 mmol/L). All patients received a single ^18^F-FDG injection (373 ± 28 MBq), followed by a simultaneous ^18^F-FDG PET/MR scan. Imaging studies were acquired with a Biograph MR system (Siemens Healthcare) with a 16-channel head and neck surface coil and 3 or 4 12-channel body coils; the number of body coils used depended on the height of the patient. These coils were combined to form a multichannel whole-body coil using total imaging matrix technology. ^18^F-FDG PET/MR imaging began a mean of 60 ± 1.2 min after the ^18^F-FDG injection. Co-acquired sequences were started along with PET acquisition from the level of the mid-thigh and moved toward the head and following from the thighs toward the feet. The bed position in the thighs, pelvis, and neck was acquired during shallow free breathing. In the upper abdomen and thorax, it was acquired during expiration breath holding as previously described [19]. Different sequences were used to cover the whole body: T2 short tau inversion recovery (STIR) and T1 turbo spin-echo in the coronal plane, diffusion-weighted imaging (DWI), and T2 half Fourier single-shot turbo spin-echo in the axial plane.

Lesion STIR positivity was defined by the presence of lesion hyperintensity. The STIR sequence analysis refers to the thyroid bed, lymph nodes, bones, and other parenchyma, such as the lungs and liver.

PET data were obtained together with MR and emission data were corrected for random, dead time, scatter, and attenuation. PET/MR images were evaluated by consensus agreement of four readers, two radiologists (C.C. and V.R. with more than 10 years of experience in body MR) and two nuclear medicine physicians (C.N. and E.N. with more than 10 years of experience); these expert readers showed an excellent level of inter-reader agreement for all parameters.

The apparent diffusion coefficient was evaluated and obtained through the images of the DWI sequences. Specifically, a log-linear regression from the DWI data was performed and sent to the image archiving system. Lesion detection and characterization were assessed on either T2w or DWI. A final assessment of positive MR was made considering all sequences in terms of malignancy based on the morphological T2-weighted features and the presence of restricted diffusivity in the DWI images and both needed to be positive on the same lesion. Both foci with abnormal uptake of ^18^F-FDG, which corresponded to tissue abnormalities on the MR, such as tissue masses or lymph nodes, and radiotracer accumulation, which did not correspond to normal morphological structures, were considered positive [19,20,21].

### 2.3. Follow-Up

All patients underwent a complete clinical and hematological follow-up every 6 months. Recurrence of disease was defined as an increase in the Tg level (≥2 ng/mL) on L-thyroxine and/or positive high-resolution neck ultrasound and confirmed by the Tg level of L-thyroxine, ^131^I uptake in patients needing further empiric ^131^I therapy undergoing a post-therapeutic WBS, and/or positive histology in the surgical specimen [4]. The date of recurrence or the most recent office visit was recorded.

### 2.4. Statistical Analysis

Continuous data are expressed as the mean ± standard deviation and categorical data as a percentage. Comparison between groups was performed with an unpaired t test or chi-square test, as appropriate. Survival analysis was performed by univariate Cox proportional hazard regression analysis. A *p <* 0.05 was considered statistically significant. Event-free survival curves were obtained by the Kaplan–Meier method and compared with the log-rank test.

## 3. Results

None of the patients potentially eligible for the study refused to participate and all patients were able to sustain the procedure. Therefore, 55 consecutive patients (29 women, 16 men; mean age 45 ± 16 years) underwent a simultaneous whole-body ^18^F-FDG PET/MR scan. In total, 19 out of 55 patients in this study with negative serum Tg and negative ^18^F-FDG PET/MR were monitored during follow-up. The clinical motivation for performing this type of imaging was the suspicion of biochemical recurrence according to the serum Tg-antibody (Tg-Ab) levels, or structural recurrence with neck lesions on the ultrasound. The patients with positive findings were referred for case-specific therapy and, afterwards, monitored during follow-up. During the follow-up (mean 42 ± 27 months, range 6–102 months), 29 patients (53%) had recurrence of the disease (mean 33 ± 28 months, range 1–102 months), 12 of them had positive ^18^F-FDG PET findings, and 8 were concordant positive PET/MR.

**Figure 1:**
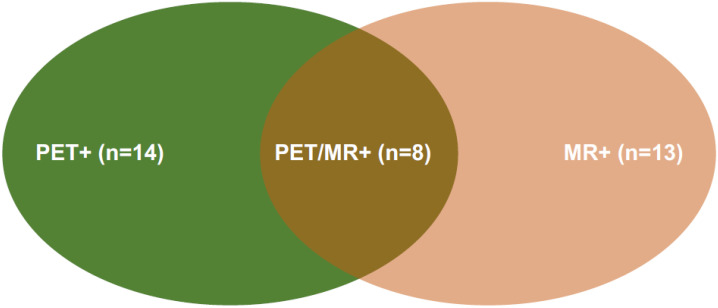
Venn diagram: 14 patients were PET+ and 13 patients MR+; the overlap of these groups is represented by 8 patients.

The baseline clinical characteristics and imaging findings in the overall patient population are summarized in Table 1. In total, 19 patients were STIR positive: 8 on neck and bone and 11 on other parenchyma, such as the lungs and liver. In total, 15 patients demonstrated DWI-positive lesions while only 13 patients were MR-positive (i.e., showing both STIR and DWI positive). Fourteen patients were positive on ^18^F-FDG PET imaging. The overlap of these groups is represented by eight patients (Figure 1). The percentage of patients with pathological findings on the STIR, DWI, and MR sequences was significantly higher (*p <* 0.05) in patients with DTC recurrence as compared to those without. The prevalence of patients with serum Tg level ≥2 ng/mL and positive ^18^F-FDG PET was higher in those with DTC recurrence compared to those without (*p <* 0.001 and *p <* 0.005).

In the Cox univariate regression analysis, age, serum Tg level ≥ 2 ng/mL, positive STIR, and positive PET were significant predictors of DTC recurrence (Table 2) while in the Cox multivariate regression analysis, only serum Tg level ≥ 2 ng/mL was a significant predictor of DTC recurrence (Table 3).

**Figure 2:**
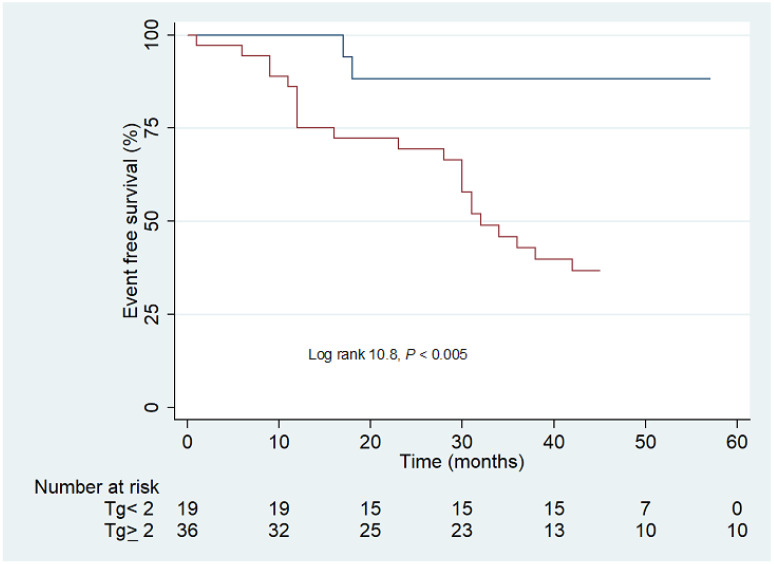
Kaplan–Meier survival analysis according to serum Tg levels. The navy line indicates patients with Tg < 2 ng/mL and the red line patients with Tg ≥ 2 ng/mL.

Kaplan–Meier survival analyses showed that patients with serum Tg level ≥ 2 ng/mL had poorer outcomes compared to those with serum Tg level < 2 ng/mL (Figure 2).

**Figure 3:**
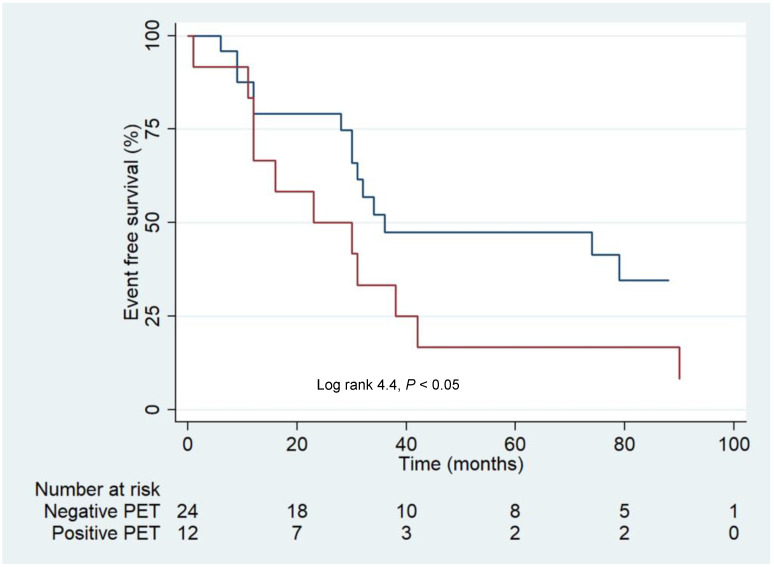
Kaplan–Meier survival analysis according to the PET findings in the subgroup of 36 patients with Tg level ≥ 2 ng/mL. The navy line indicates patients with negative PET and the red line patients with positive PET.

Survival analysis performed in the subgroup of 36 subjects with Tg level ≥ 2 ng/mL revealed that patients with positive PET had a worst outcome compared to those with negative PET (Figure 3).

**Figure 4:**
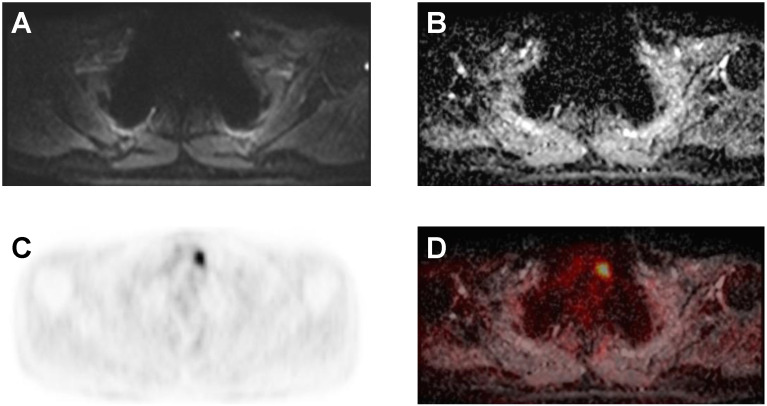
Example of a lesion detected in the right digastric jugular space at MR with inhomogeneous hyperintensity in the T2 sequences (**A**), restricted diffusivity in the DWI sequences (**B**), and increased uptake of ^18^F-FDG on PET (**C**) and PET/MR (**D**).

A representative example of a case demonstrating discordant imaging findings is depicted in Figure 4.

## 4. Discussion

The results of our study suggest that age, serum Tg level ≥ 2 ng/mL, positive STIR, and positive ^18^F-FDG PET are predictors of DTC recurrence. Moreover, in patients with serum Tg level ≥ 2 ng/mL, a positive ^18^F-FDG PET was able to further stratify patients. In addition, the high prevalence of ^18^FDG-PET/MR positivity in patients with disease recurrence could be related to higher serum Tg levels. These findings suggest that higher serum Tg levels indirectly denote a greater tumor burden.

^18^F-FDG PET/CT, which combines the advantages of metabolic and morphologic imaging, allowing for anatomic localization of focal metabolic activity, is an important tool for the evaluation of patients with DTC, mainly in those with elevated Tg and negative WBS, and has a potential role in personalized therapy of DTC [6,7,8,9,10]. However, supplementary imaging modalities are occasionally required. MR is the primary imaging modality for soft tissue evaluation due to its excellent soft tissue contrast and additional functional imaging capabilities. The hybrid PET/MR imaging system allows a reduction in the radiation dose, with very promising results in oncological patients; therefore, several authors have dealt with the topic of PET/MR in the evaluation of tumoral diseases [22,23,24,25,26,27,28].

Song et al. [16] explored the clinical value of ^18^F-FDG PET/MR in a head-to-head comparison with PET/CT in loco-regional recurrent and metastatic cervical lymph nodes of DTC patients after comprehensive treatment. Compared with PET/CT, PET/MR showed better detection rates, and higher diagnostic sensitivity and accuracy. For the same lesion, SUV and diameters measured by PET/MR and PET/CT were consistent and had a significant correlation. The authors concluded that the addition of local PET/MR after whole-body PET/CT may be recommended to provide more precise diagnostic information and scope for surgical resection without additional ionizing radiation. Jentzen et al. [17] compared the quantitative performance of ^124^I PET/MRI and ^124^I PET/CT. Their study showed a comparable quantitative PET performance between the two aforementioned modalities, further suggesting that PET/MRI can be reliably used for the evaluation of thyroid cancer in the initial staging and follow-up settings. Although the current published literature is relatively sparse, there are potential benefits of a hybrid modality of PET/MR imaging. With the increasing number of PET/MR scanners in clinical use and ongoing research, the role of PET/MR imaging in the management of head and neck cancer is likely to become more evident in the near future [18].

The role of ^18^F-FDG PET/MR in the evaluation and follow-up of patients with DTC has been previously addressed. In the study by Vrachimis et al. [21], an optimal agreement was found between ^18^F-FDG PET/MR and PET/CT on a patient basis in DTC, but PET/MR was less sensitive in the detection of lung metastases. In another study, Vrachimis et al. [22] demonstrated that ^18^F-FDG PET/CT was more sensitive than ^68^Ga-DOTATATE PET/MRI in the evaluation of RAI-refractory DTC, mostly because of its excellent ability to detect lung metastases. In the evaluation of extrapulmonary lesions, ^68^Ga-DOTATATE PET/MRI was more sensitive and ^18^F-FDG PET/CT more specific. Furthermore, DWI did not provide additional information and cannot replace ^18^F-FDG PET/CT for postoperative monitoring of patients with suspected RAI-refractory DTC. Gross et al. [19] showed that MR imaging is a sensitive and accurate technique for the detection of DTC, particularly papillary carcinoma, metastatic to cervical lymph nodes. However, the lower specificity of this modality precludes its use as a screening tool.

Hempel et al. [20] evaluated combined ^18^F-FDG PET/CT and MR in the detection of local recurrence and distant metastases in patients with elevated Tg levels, negative ultrasound, and WBS. They found a complementary role of the two modalities, suggesting a combined approach in high-risk DTC patients. Klain et al. [29] sequentially performed ^18^F-FDG PET/MR and PET/CT in 40 consecutive patients with an aggressive histology, absence of radioioderine uptake in neoplastic foci, and absence of imaging abnormalities in patients with an elevated serum Tg level. The authors compared the diagnostic performance of these two imaging methods, reporting that ^18^F-FDG PET/MR was positive in 11 patients and ^18^F-FDG PET/CT in 10 patients, with 33 and 30 tumor foci detected, respectively. They concluded that ^18^F-FDG PET/MR offered only minor improvements over ^18^F-FDG PET/CT, thus it might be performed in selected patients who can cooperate, including those for whom radiation exposure should be minimized and those for whom MR imaging is indicated. An alternative is to first perform ^18^F-FDG PET/CT and then, whenever needed, MR imaging. Li et al. [30] reported the diagnostic performance of ^18^F-FDG PET/MR in detecting recurrent or metastatic disease in patients with DTC who had increased Tg levels but a negative WBS scan. Of the 29 patients studied, ^18^F-FDG PET/MR was true positive in 18 patients, with significant differences in the serum Tg levels between the ^18^F-FDG PET/MR-positive and ^18^F-FDG PET/MR-negative patient groups (*p* = 0.04). A Tg level of 2.4 ng/mL was the optimal cut-off value for predicting the ^18^F-FDG PET/MR results.

## 5. Conclusions

In this investigation age, serum Tg level ≥2 ng/mL, positive STIR, and positive ^18^F-FDG PET were significant predictors of DTC recurrence. However, the serum Tg level was the only independent predictor of DTC. These findings suggest that the optimal time point for ^18^FDG-PET/MR imaging is probably when the serum Tg level is ≥2 ng/ml. Even if the superiority of PET/MR over both functional and morphological imaging methods has not yet been proven, there are certainly particular clinical conditions that make some aspects fundamental, such as the study of the pediatric population, nervous system, and soft tissue, and the absence of radiation. However, there are also limitations, such as the long acquisition times, claustrophobia, high cost, and limited availability of this hybrid system in the area. We are still a long way from giving this imaging method a diagnostic role in certain pathologies, but the potential is certainly there. Thus, further studies in a larger patient population are needed to understand the additional value of ^18^F-FDG PET/MR in patients with DTC.

## Figures and Tables

**Table 1:** Baseline characteristics in the overall population according to recurrence.

	All Patients (*n* = 55)	Without Recurrence (*n* = 26)	With Recurrence (*n* = 29)	*p* Value
Age (years)	45 ± 16	41 ± 15	47 ± 17	0.16
Age ≥ 45 years, *n (%)*	23 (42)	8 (31)	15 (52)	0.12
Male gender, *n (%)*	16 (29)	8 (31)	8 (28)	0.79
Papillary histology, *n (%)*	40 (73)	22 (85)	18 (62)	0.06
^131^I therapy (*n*)	2.4 ± 1.5	2.4 ± 1.4	2.3 ± 1.6	0.71
Tg at time of PET/MR (ng/mL)	98 ± 329	4.6 ± 10	181 ± 441	0.04
Tg ≥ 2 ng/mL, *n (%)*	36 (65)	9 (35)	27 (93)	0.001
Stage > 1, *n (%)*	10 (18)	3 (12)	7 (24)	0.26
Positive STIR, *n (%)*	19 (35)	5 (19)	14 (48)	0.02
Positive DWI, *n (%)*	15 (27)	3 (12)	12 (41)	0.01
Positive MR, *n (%)*	13 (24)	3 (12)	10 (34)	0.04
Positive PET, *n (%)*	14 (25)	2 (8)	12 (41)	0.004
Follow-up (months)	42 ± 27	51 ± 22	33 ± 28	0.009

Data are presented as the mean ± SD or number and percentage (%). Tg: thyroglobulin; STIR: short tau inversion recovery; DWI: diffusion-weighted imaging; MR: magnetic resonance; PET: positron emission tomography.

**Table 2:** Cox univariate regression analysis to predict DTC recurrence.

	Hazard Ratio (95% CI)	*p* Value
Age	1.023 (1.001–1.046)	0.04
Male gender	1.060 (0.466–2.411)	0.89
Papillary histology	0.830 (0.334–2.061)	0.69
Number of ^131^I therapy	1.280 (0.951–1.723)	0.10
Tg at time of PET/MR	1.001 (1.000–1.001)	0.12
Tg ≥ 2 ng/mL	0.130 (0.030–0.555)	0.006
Stage >1	2.137 (0.850–5.370)	0.11
Positive STIR	0.471 (0.224–0.991)	0.04
Positive DWI	0.500 (0.235–1.063)	0.07
Positive MR	0.584 (0.267–1.277)	0.18
Positive PET	0.347 (0.163–0.737)	0.006

Data are presented as the mean ± SD or number and percentage (%); Tg: thyroglobulin; STIR: short tau inversion recovery; DWI: diffusion-weighted imaging; MR: magnetic resonance; PET: positron emission tomography.

**Table 3:** Cox multivariate regression analysis to predict DTC recurrence.

	Hazard Ratio (95% CI)	*p* Value
Age	1.017 (0.995–1.039)	0.13
Tg ≥ 2 ng/mL	0.142 (0.032–0.621)	0.01
Positive STIR	0.937 (0.326–2.691)	0.90
Positive PET	0.647 (0.210–1.996)	0.45

## Data Availability

The data presented in this study are available on request from the corresponding author. The data are not publicly available due to privacy restrictions.
